# Effects of a 24-week multicomponent training program on functional capacity, persistent symptoms, body composition, and physical activity in patients significantly affected by COVID-19: the COVID-19 and REhabilitation study (CORE-study)—randomized clinical trial

**DOI:** 10.3389/fspor.2025.1549132

**Published:** 2025-09-22

**Authors:** Angelica Danielevicz, Mabel Diesel, Carla Elane Silva dos Santos, Maria Eduarda de Moraes Sirydakis, Paulo Urubatan Gama de Melo, Marina Isolde Constantini, Fernanda Hansen, Aline Mendes Gerage, Cintia de la Rocha Freitas, Cassiano Ricardo Rech, Fernanda Rodrigues Fonseca, Rosemeri Maurici, Rodrigo Sudatti Delevatti

**Affiliations:** ^1^ Sports Center, Federal University of Santa Catarina, University Campus, Florianópolis, Santa Catarina, Brazil; ^2^ Health Sciences Center, Federal University of Santa Catarina, Florianópolis, Santa Catarina, Brazil; ^3^ Health Sciences Center/NUPAIVA, Federal University of Santa Catarina, Florianópolis, Brazil

**Keywords:** rehabilitation, physical exercise, COVID-19, physical capacity, SARS-CoV-2

## Abstract

**Background:**

COVID-19 can lead to reduced functional capacity, loss of muscle mass, and lasting and persistent symptoms, resulting in reduced physical activity.

**Objective:**

To evaluate the effects of a multicomponent training on functional capacity, persistent symptoms, body composition, pulmonary function, and physical activity levels in patients significantly impaired by SARS-CoV-2.

**Methods:**

The participants were randomly assigned (1:1) to either the intervention group (IG), which received multicomponent training (balance/aerobic/resistance), or the control group (CG). Functional capacity [6 min walk test (6MWT)—primary outcome, sit and reach, sit-to-stand, timed up and go], persistent symptoms (dyspnea, fatigue, post-COVID functional status, frailty), body composition (dual-energy x-ray absorptiometry and bioimpedance), pulmonary function, and physical activity levels (accelerometry) were evaluated at baseline and after 24 weeks. Generalized estimating equations were used, with the significance level set at *α* = 0.05. Outcomes were analyzed by intention-to-treat (ITT) and per-protocol (PP) approaches. Effect sizes were calculated from the mean difference between groups of changes between pre- and post-intervention.

**Results:**

Forty participants [age = 52.00 (12.93) years, 19 women] were included. The primary outcome 6MWT showed improvement in both groups in the ITT analysis (IG: 35.5 m, 95% CI: −3.0 to 74.1; CG: 37.4 m, 95% CI: −5.26 to 80.2) and in the IG (87.6 m, 95% CI: 50.6–124.4) in the PP analysis. The IG showed a reduction in mental fatigue (−1.7 points, 95% CI: −0.5 to 3.5) and general fatigue (−6.5 points, 95% CI: −9.4 to −3.5) in our ITT analysis. The IG also revealed improvement in timed up and go test (−1.6 s, 95% CI: −2.6 to −0.6), mental fatigue (−2.0points, 95% CI: −3.6 to 0.7), general fatigue (−6.4points, 95% CI: −11.0 to −1.6), and a protective effect against increased body fat in PP analysis.

**Conclusion:**

This program was effective in improving fatigue in patients previously significantly affected by COVID-19.

## 1 Introduction

The manifestation of COVID-19 symptoms is not homogeneous, with some infected individuals showing no symptoms while others rapidly progress to severe and critical cases (1). Particularly among hospitalized individuals, the disease is exacerbated by physical inactivity, leading to adverse impacts on functional capacity (2, 3) and body composition, especially in muscle quantity and function (4–6).

Following the acute phase of COVID-19, many individuals experience a syndrome known as post-COVID-19 condition, and the number of people with late sequelae remains unknown (7). This condition is characterized by substantial functional impairment (2, 3) and may encompass long-lasting symptoms such as dyspnea and fatigue, accompanied by diminished exercise tolerance and functional restrictions (8–11). Fatigue stands out as the most prevalent symptom in both hospitalized and non-hospitalized patients, persisting up to 2 years postinfection (12–15). This symptom may have as one of its origins the muscle damage caused during the infection, which may result from mitochondrial changes, inflammation, capillary injury in muscle biopsies, and reduced energy supply (16–18). The physical and functional limitations observed in post-COVID-19 condition perpetuate a vicious cycle, wherein reduced physical activity (PA) levels are associated with a lower functional capacity and so on.

In this context, exercise intolerance, primarily driven by persistent dyspnea and fatigue (16), emerges as a multifaceted phenomenon influenced by both physical and psychological factors (19). Given that reduced functional capacity is associated with a higher risk of mortality in clinical populations (20, 21) and a decline in activities of daily living among the elderly (22), there is an urgent public health need to reduce COVID-19 symptoms both in the acute phase and persistent symptoms and to enhance the functional capacity of survivors (3, 23, 24). Physical exercise rehabilitation plays a pivotal role in addressing these challenges (25, 26).

Recent studies have begun to uncover the benefits of post-COVID rehabilitation models that emphasize exercise (26–32). These models have shown promise in improving various outcomes such as fatigue, functional capacity, strength, muscle cross-sectional area, and muscle quality (27–30). A recent meta-analysis concluded that respiratory training- and exercise-based rehabilitation interventions are effective in improving functional capacity (6 min walking test, dyspnea) in post-COVID-19 conditions. However, there is moderate and low certainty of evidence, respectively. Regarding fatigue, the evidence was limited and could not be synthesized (33). Additionally, body composition is an outcome little evaluated in randomized clinical trials to date, despite its involvement in the acute phase of the disease and possibly in the post-COVID-19 condition (26, 31, 32).

In this regard, most of the evidence from randomized controlled trials to date reports brief interventions, lasting up to a maximum of 16 weeks, often delivered in alternative formats such as semi-supervised, tele-supervised, or home-based (26, 32–34). However, there remains a scarcity of randomized controlled trials investigating multicomponent training strategies—those that combine different physical abilities—to address the complex, limiting symptoms and functional limitations often observed in post-critical COVID-19 cases.

In addressing this research gap, our study examined the impact of a multicomponent training regimen on functional outcomes, body composition, and persistent symptoms among patients significantly affected by COVID-19. Additionally, we assessed physical activity levels and sedentary behavior (SB) before and after the intervention. The hypothesis is that multicomponent training will be superior to a control procedure, involving physical activity recommendations, in improving functional capacity, pulmonary function, persistent symptoms, body composition, and physical activity levels in patients post-COVID-19 infection.

## 2 Methods

### 2.1 Study design

This was a single-center, single-blinded, parallel-group, randomized, controlled trial conducted at the Federal University of Santa Catarina (UFSC), Florianópolis, Brazil, between November 2021 and April 2023. The complete protocol can be found in Delevatti et al. (35). This study was approved by the Human Research Ethics Committee of the institution of origin (Protocol 4,909,599), pre-registered in the Brazilian Registry of Clinical Trials (RBR-10y6jhrs), and is nested within a larger controlled trial, multicentric, with different training/rehabilitation programs, called Recovery Trial. The manuscript was reported according to the Consolidated Standards of Reporting Trials (CONSORT) guidelines (36). All participants provided oral and written informed consent.

### 2.2 Participants

Women and men (≥18 years old) who had previously been admitted to the UFSC University Hospital with moderate to critical COVID-19 (37) or who were not hospitalized but experienced chronic postinfection fatigue [score >4 on the Chalder scale (38)] participated in the study.

All eligible and interested patients who wished to participate in the study underwent a prior medical screening which included clinical evaluation by physicians. The eligibility criteria for the study were as follows: minimum age of 18 years; discharge from the hospital or completion of the acute phase of the disease at least 6 weeks prior; and general respiratory, functional, and cognitive stability. More details about the eligibility criteria can be found in Delevatti et al. (35). Individuals who had previously participated in a rehabilitation or physical exercise program or who were not available to participate in the proposed intervention were excluded from the study.

Participant selection was non-probabilistic and voluntary. The participants were recruited through medical screening at the University Hospital—UFSC or through contact through online and television advertising materials. All participants provided oral and written informed consent after the objectives, procedures, and risks of the study had been explained.

### 2.3 Randomization and blinding

The participants were randomized (www.randomizer.org) in permuted blocks of 4–6, stratified by sex, into either the intervention group (IG) or the control group (CG) in a 1:1 ratio. A computer-generated random number sequence was created by an independent researcher. After baseline measurements, the participants were assigned consecutive numbers, which were forwarded to an external data manager, who subsequently returned the corresponding allocation to the study’s exercise providers. Blinding of the participants and exercise providers was not possible after group allocation. However, the study's care providers (exercise and control procedure) did not participate in treatment action assessment and data analysis or interpretation.

All assessments were conducted by the same evaluators at baseline and after 24 weeks. All evaluators were experienced in collecting the outcomes and were blinded to group allocation; moreover, the intervention team did not participate in outcome assessments. Unfortunately, however, blinding of the statistical analysis was not possible.

### 2.4 Clinical data

For the participants who agreed to participate, a complementary medical history was administered during the medical screening, containing sociodemographic and general health information, along with a clinical history related to the period of COVID-19 infection, such as type of hospitalization, duration of hospitalization, and use or non-use of mechanical ventilation.

### 2.5 Intervention

The intervention was a 24-week supervised program divided into two phases. Training was performed twice a week in Phase 1 and thrice a week in Phase 2 (∼70 min/session). The 24 weeks were composed of 1 week of familiarization and two mesocycles of 5 weeks in Phase 1, 1 regenerative week, and three mesocycles of 4 weeks in Phase 2. The program had a multicomponent structure, with balance (15 min/session), resistance training (15–20 min/session), and aerobic training (25 min/session) in Phase 1 and resistance training (20–25 min/session) and aerobic training (25 min/session) in Phase 2. The progression strategies adopted in Phase 1 were an increase in volume in resistance and aerobic training and an increase in complexity in balance training. In Phase 2, the progression aimed to increase the intensity of strength and aerobic training, while balance training was discontinued.

**Figure 1:**
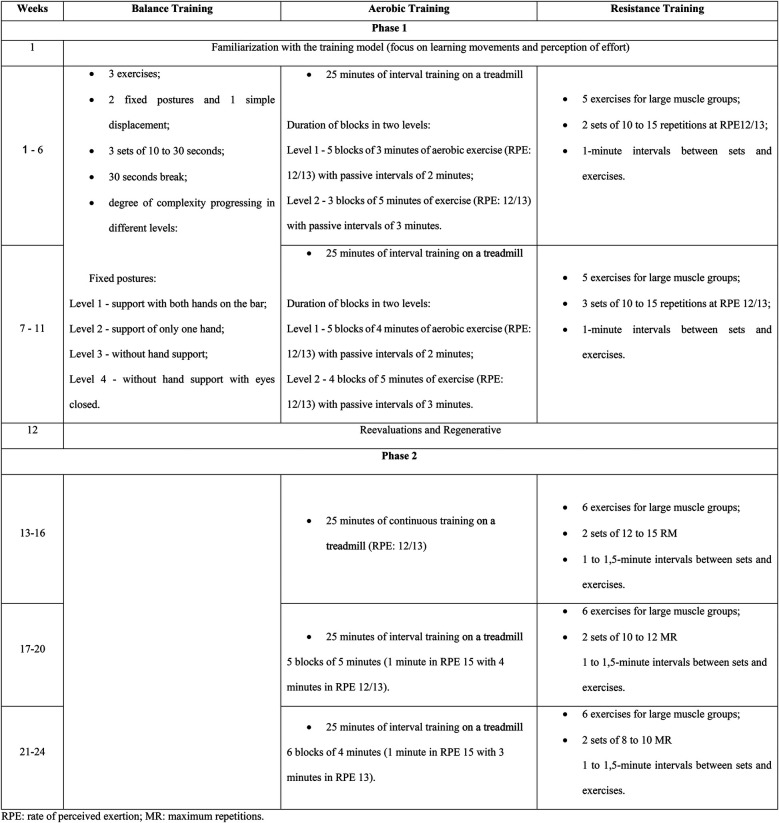
Structuring of training sessions throughout the rehabilitation program (CORE-study). RPE, rate of perceived exertion; MR, maximum repetitions.

The order of the main parts (resistance and aerobic) was alternated over the weeks with the aim of not prioritizing one of the components and to serve as a motivational factor. Balance training was discontinued in Phase 2, but if trainers identified a patient who still required this training, this was included individually at the beginning of the session. In Phase 2, resistance training consisted of training with machines and weights, while in Phase 1, it was just body weight and resistance bands. Details of the intervention, such as specific exercises and progression strategies, are presented in Figure 1.

The sessions happened in the Rehabilitation Center of the Sports Center, UFSC. Three instructors with previous experience in exercise prescription supervised the intervention, and physical education students helped with the general running of the sessions. The instructor–patient ratio in the sessions was 1:2.

### 2.6 Adherence

The adherence was assessed by the frequency of participation in the training sessions. When a participant in the intervention group had two or more absences, they were contacted to determine the reason for their absence and to offer assistance if necessary.

### 2.7 Control procedures

The patients allocated to the control group received recommendations and guidance on physical activity and sedentary behavior in a face-to-face meeting lasting approximately 1 h. The information was presented by one of the professors responsible for the program and taken from two chapters of the Brazilian Guide to Physical Activity (39) and was directed to the age group of each patient (adult or elderly). One of the chapters, titled “Understanding Physical Activity,” primarily focuses on understanding the domains of physical activity, intensity levels, and the physical capacities that should be prioritized. The other chapter is age-specific, presenting the main recommendations regarding the amount of weekly practice time, appropriate intensity levels, possible activities, and the physical capacities that should be prioritized—for example, aerobic and strength activities for adults and aerobic, strength, and balance activities for older adults. The patients received copies of the chapters presented and had the opportunity to clarify possible doubts about the topics addressed.

### 2.8 Load and safety monitoring

The external and internal training load, peripheral O_2_ saturation (SpO_2_), heart rate (HR), blood pressure, and capillary glycemia were evaluated. The internal load was assessed using the rate of perceived exertion (RPE) of the session, measured on the Borg CR10 scale adapted by Foster et al. (40). The external load from resistance training was assessed through repetitions in the sit-and-stand exercise during Phase 1 and through weights in the leg press exercise during Phase 2. For aerobic training, the external load was evaluated through distance covered and speed on the treadmill. SpO_2_ was evaluated before, during, and after all exercise sessions, and the participants did not start or had the session interrupted immediately if the oximetry showed values <90%. Blood pressure was also measured before all exercise sessions following the procedures described by Barroso et al. (41). Capillary blood glucose collections were performed primarily for safety, in patients with diabetes, adopting the cutoff point to start the session, with values between 100 and 250 mg/dL.

### 2.9 Adverse events

The patients were monitored throughout the program for the occurrence of adverse events using a standardized form. This form has questions about the general well-being of the patient, as well as symptoms, pain, and other adverse events associated with physical exercise or not. In the IG, this information was collected weekly, while for the CG, this form was completed by video call every 6 weeks and at the end of the study period.

We classified the severity of adverse events as follows: catastrophic, for events resulting in death; major, for events causing permanent harm, including loss of function; moderate, for events resulting in semipermanent harm lasting from 3 days up to 1 year; and minor, for events causing no permanent harm and lasting <3 days (42).

### 2.10 Outcomes

All outcomes were evaluated before the start of the intervention and after 24 weeks.

#### 2.10.1 Functional capacity

The primary outcome is the change in the distance covered in the 6 min walk test (6MWT), as it is the most representative outcome of the general functionality. The 6MWT was performed according to the American Thoracic Society (43) in a predetermined space of 30 m. Every 2 min, the values of HR, peripheral oxygen saturation (SpO_2_), and perception of central and peripheral exertion by the Borg CR10 scale, adapted by Foster et al. (40), were recorded. Two attempts were made with an interval of at least 15 min, and the longest distance covered was adopted.

The sit-to-stand (STS) test was performed according to the Rikli and Jones (44) battery, measuring the number of repetitions performed in 30 s. The timed up and go (TUG) test was performed in two attempts for each of the speeds, maximum (TUG-m) and usual (TUG-u), with a 1 min interval between each attempt, adopting the shortest time for each speed (45). Flexibility was evaluated by the sit and reach test (46), using the Wells bank as an instrument, adopting the highest value achieved in two attempts.

#### 2.10.2 Persistent symptoms

Four different scales involving functional status were applied: Modified Medical Research Council (MMRC) (47), Chalder Fatigue Scale (38), Post-COVID-19 Functional Status (PCFS) (48), and Tilburg Frailty Indicator (49).

#### 2.10.3 Body composition, phase angle (PhA), and anthropometry

Body composition was assessed using computerized densitometry by dual-energy x-ray absorptiometry (DXA) with a Hologic® instrument (Discovery Wi Fan-beam S/N 81593, Hologic, Inc., Bedford, MA, USA) calibrated and used following the manufacturer's recommendations. PhA measurement was evaluated using multifrequency octapolar bioimpedance (InBody® 720, Biospace, Los Angeles, CA, USA). The PhA was calculated using the following equation: PhA = arctan (*X*c/*R*) × (180 /*π*) (50). To determine the body mass index (BMI in kg/m^2^), body mass was measured using the previously mentioned bioimpedance device, and height was measured using a stadiometer (Alturaexata®, with 1 mm precision). The waist was measured using a flexible and inelastic measuring tape (Cescorf®, with a precision of 1 mm), and the waist-to-height ratio (WHR) was determined.

All measurements were always taken in the morning after at least 4 h of fasting. All participants were instructed to avoid physical exercise the day before the assessment; to abstain from alcoholic, caffeinated, or diuretic drinks in the 48 h prior to assessment; and, in the case of women, to not be menstruating. The participants were also instructed to wear gym clothes without zippers or metal; to be barefoot; to remove earrings, rings, or any type of adornment; and to urinate 30 min before assessment. After the assessments, everyone received a standardized snack consisting of 25 g of whole-grain crackers and a banana. Further details can be found in Delevatti et al (35).

#### 2.10.4 Physical activity and sedentary behavior

Physical activity and sedentary behavior were evaluated with GT3X+ accelerometers. The accelerometer was affixed to the right side of the hip using an elastic belt, and the participants were instructed to wear it continuously for 7 consecutive days. They were asked to remove the accelerometer only during sleep, showering, or activities involving water. Data were collected at a frequency of 30 H and analyzed in 60 s epochs. We interpreted consecutive values of zero (with a tolerance of 2 min) over ≥60 min. as a period of non-use and excluded them from the analysis (51). The data were only considered valid when the participant had used the accelerometer and had accumulated a minimum number of records over 4 days of use during the week, including one weekend day (10 h/day). The mean values at each PA intensity and mean SB were calculated using the cutoff points by Freedson et al. (52), considering SB as 0–99 counts/min, light physical activity as 100–1,951 counts/min, and moderate to vigorous physical activity as ≥1,952 counts/min, using the vertical axis. These cutoff points were applied for the adult population. The data were analyzed with the ActiLife software (Actigraph v.6.12.1). All data were analyzed as minutes/day to adjust for the number of days when the device was used.

#### 2.10.5 Pulmonary function

Pulmonary function assessment was carried out following the guidelines established by the American Thoracic Society and the European Respiratory Society for the single-breath carbon monoxide uptake in the lung (53). The diffusion lung capacity for carbon monoxide (DLCO, mL/min/mmHg), alveolar volume (VA, L), and carbon monoxide transfer coefficient (KCO, mL/min/mmHg/L) were measured using the Vmax® system (VIASYS Respiratory Care Inc., USA). DLCO, VA, and KCO values were also expressed as percentages of the predicted values, according to Guimarães et al. (54).

### 2.11 Statistical analyses

Sample size was calculated *a priori* considering the distance covered in the 6MWT as the primary outcome based on the study of Liu et al. (55). This study showed an intragroup and intergroup change after the 50 m intervention in favor of the intervention group, which is the expected clinical difference in the study. The sample size calculation was conducted for a repeated measures ANOVA (within-between interaction), using the *f* effect size (value 0.35), a two-tailed *α* level of 0.05, a repeated measures correlation of 0.5, and a desired statistical power of 80% (*β* = 0.20). First, the effect size *d* was calculated using the means and standard deviations reported in Liu et al. (55). Then, this *d* value was converted into *f* for use in G*Power. The estimated required sample size was 22 individuals per group. However, accounting for a potential sample loss of 30%, the recruitment target was set at 30 participants per group.

The continuous sample characterization variables were tested for normality and homogeneity using the Shapiro–Wilk and Levene tests, respectively. Continuous variables are presented by mean and standard deviation or by median and interquartile range. The categorical variables characterizing the sample were presented by absolute (sampled *n*) and relative (%) frequencies. For comparison between groups, the independent *t*-test or its non-parametric corresponding test was used for continuous data and Fisher's exact test for categorical data.

Outcomes were presented by mean and standard error. Analysis by generalized estimation equations was used, adopting the Bonferroni *post hoc* test. The generalized estimating equation method with normal distribution, logarithmic link function, robust estimate of covariance matrix, and interdependent working correlation matrix structure was used to assess differences both between and within groups, using “group” and “time” as factors across all outcomes. Outcomes were analyzed by intention-to-treat (ITT) and per-protocol (PP) analyses. For the PP analysis, the patients who completed at least 70% of the proposed sessions in the IG and those who attended the 24-week evaluations in the CG were included. Participation was monitored through direct supervision. For ITT analysis, the pre-intervention values of all randomized patients and the post-intervention values of all patients assessed at that time were maintained. For missing post-intervention data, a maximum likelihood estimate was used for imputation. This method estimates missing data based on the distributions of observed data to predict the most likely values of the missing data. The adopted significance index was 0.05. Effect sizes (ES) were calculated from the mean difference between groups of changes between pre- and post-intervention (*Δ*IG − *Δ*CG), divided by the pooled baseline standard deviation, as described by Morris (56). Values were classified as small (0.20 ≤ d < 0.50), medium (0.50 ≤ *d* < 0.80), and large (*d* ≥ 0.80). The statistical analysis was carried out using Statistical Package for the Social Sciences (SPSS), version 22.0.

## 3 Results

**Figure 2:**
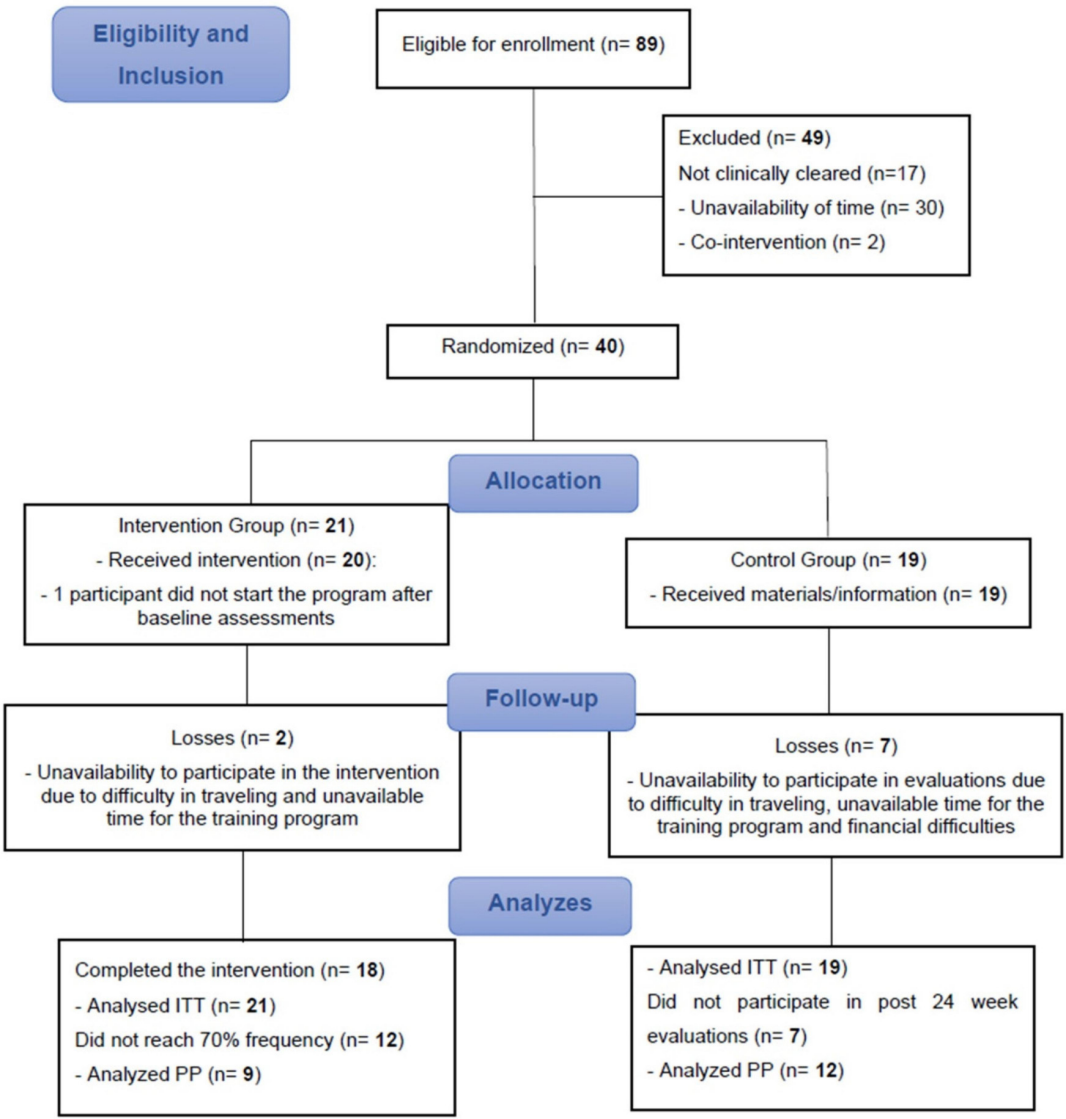
Flow of the participants through the study.

A total of 89 participants volunteered to take part in the study, of whom 49 did not meet the inclusion criteria or refused to participate after being duly informed of the objectives and procedures. Thus, 40 participants were randomized, 21 in the IG and 19 in the CG. During study follow-up, three participants withdrew from the IG, and five participants from the CG did not participate in the 24-week evaluations for personal reasons (Figure 2).

There were no between-group differences in sociodemographic data, presence of chronic diseases, types and periods of hospitalization, and symptoms. In general, of the 40 participants (21 male and 18 female), 31 (77.5%) underwent hospitalization in the acute phase of the disease, and 21 (52.50%) required mechanical ventilation. The most common symptom, both in the acute and the post-phase, was fatigue, reported by 75% of participants at both times (Table 1). The mean internal and external loads for each phase, as well as the overall 24-week period, are presented in Table 2.

**Table 1:** Baseline characteristics of the participants.

	IG (*n* = 21)	CG (*n* = 19)	*p-*value
Demographics			
Age (years)	50.7 ± 14.2	53.4 ± 11.6	0.531
Sex (M/F)	11/10	10/9	>0.999
Time post-COVID (dias)	384.7 ± 171.1	376.6 ± 195.1	0.889
Clinical features			
Hypertension, *n* (%)	5 (23.8)	7 (36.8)	0.494
Diabetes mellitus, *n* (%)	5 (23.8)	3 (15.8)	0.698
Dyslipidemia, *n* (%)	4 (19.0)	2 (10.5)	0.664
Former smokers, *n* (%)	5 (23.8)	7 (36.8)	0.949
Pulmonary disease, *n* (%)	4 (19.0)	2 (10.5)	0.664
Hospitalization			
ICU, *n* (%)	13 (61.9)	13 (68.4)	0.748
Nursery, *n* (%)	4 (19)	1 (5.3)	0.345
Without hospitalization, *n* (%)	4 (19)	5 (26.3)	0.712
Mechanical Ventilation, *n* (%)	9 (42.9)	12 (63.2)	0.225
ICU, days	11 (13)	14 (12)	0.341
Mechanical ventilation, days	8 (18)	8 (12)	0.639
Hospital length of stay, days	15 (15)	18 (19)	0.771
Symptoms during the acute phase, *n*			
Dyspnea	17 (81.0)	13 (68.4)	0.473
Myalgia	10 (47.6)	12 (63.2)	0.360
Fatigue	14 (66.7)	16 (84.2)	0.281
Symptoms post-acute phase, n			
Dyspnea	16 (76.2)	11 (57.9)	0.314
Myalgia	11 (52.4)	11 (57.9)	0.761
Fatigue	14 (66.7)	16 (84.2)	0.281

Data described by mean and standard deviation, median (IQR), and absolute and relative frequency (%); M, male; F, female; ICU, intensive care unit; IG, intervention group; CG, control group.

**Table 2:** The mean of internal and external loads.

Parameters	Mean ± SD		
	Phase 1	Phase 2	24 weeks
External load			
Speed (km/h)	4.5 ± 1.2	3.5 ± 1.5	4.1 ± 1.2
Distance (km)	1.2 ± 0.3	1.9 ± 1.5	1.5 ± 0.8
Sit-and-stand (repetitions)	14 ± 1.4	–	14 ± 1.4
Weight—leg press (bars)	–	5.2 ± 2.0	5.2 ± 2.0
Internal load			
RPE	3.2 ± 0.9	2.3 ± 0.7	2.8 ± 0.8

### 3.1 Adherence to the exercise intervention

The mean adherence of the IG participants was 60.91% or 1.5 weekly sessions, with nine not reaching the 70% minimum frequency for the PP analysis. The adherence means of the IG participants who reached >70% session attendance and were included in the PP analysis was 82.38% or 2.1 sessions per week.

During the 24 weeks of the program, one participant experienced an acute myocardial infarction, but this event did not occur during or shortly after a training session. There were no serious adverse events related to the intervention.

### 3.2 Functional outcomes

**Figure 3:**
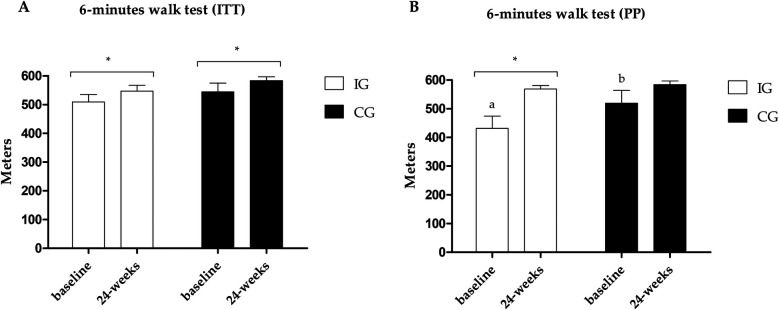
**(A)** Results of the 6MWT in the PP analysis. **(B)** Results of the 6MWT in the ITT analysis. *Different values between baseline and 24 weeks. Different letters indicate differences between groups at baseline.

The primary outcome, distance covered in the 6MWT, improved in both groups in the ITT analysis; however, in the PP analysis, only the IG improved (ES: 0.61; 95% CI: 50.6–124.4; *p* = 0.001), with differences between groups at baseline showing higher values in the CG (Figure 3). The results of the secondary outcomes are presented in Table 3. Both groups improved STS, TUG-u, and flexibility test scores in both analyses (ITT and PP), with differences between groups in the flexibility test at baseline and post-intervention showing higher values in the CG for the ITT analysis. In the TUG-m, both groups showed improvements in the ITT analysis, and only the IG showed significant improvements (ES: −0.50; 95% CI: −2.6 to −0.6; *p* = 0.029) in PP analysis, with differences between groups at baseline showing higher values in the IG.

**Table 3:** Functional capacity in the pre- and post-intervention moments—physical tests (*n* = 40).

Outcomes	Group (*n*)	Baseline	24 weeks	Mean difference (95% CI)	*p* group	*p* time	*p* interaction	ES
Intention to treat (ITT)								
STS (rep)	IG (21)	13.4 ± 0.7	15.6 ± 1.0	2.2 (0.9–3.3)	0.458	0.001	0.957	0.03
	CG (19)	14.4 ± 0.8	16.5 ± 1.2	2.1 (−0.1 to 4.3)				
TUG-m (s)	IG (21)	7.4 ± 0.5	6.7 ± 0.4	−0.7 (−1.2 to −0.1)	0.080	0.001	0.981	0.00
	CG (19)	6.5 ± 0.3	5.8 ± 0.2	−0.7 (−1.3 to −0.1)				
TUG-u (s)	IG (21)	10.3 ± 1.0	8.7 ± 0.6	−1.6 (−2.9 to −0.1)	0.177	<0.001	0.943	−0.03
	CG (19)	9.1 ± 0.5	7.6 ± 0.3	−1.5 (−2.4 to −0.5)				
Flex (cm)	IG (21)	10.4 ± 1.3^a^	13.8 ± 1.8^a^	3.4 (1.3–5.3)	0.015	<0.001	0.282	−0.28
	CG (19)	16.3 ± 2.1^b^	21.9 ± 3.0^b^	5.6 (1.9–9.2)				
Per protocol (PP)								
STS (rep)	IG (9)	13.4 ± 1.4	15.2 ± 1.2	1.8 (0.7–2.8)	0.498	<0.001	0.672	−0.08
	CG (10)	14.4 ± 0.7	16.5 ± 1.2	2.1 (1.0–3.1)				
TUG-m (s)	IG (9)	8.8 ± 1.0^a^	7.2 ± 0.5^*^^,^^b^	−1.6 (−2.6 to −0.6)	0.013	<0.001	0.029	−0.50
	CG (10)	6.2 ± 0.2^a^	5.8 ± 0.2^b^	−0.4 (−0.7 to −0.1)				
TUG-u (s)	IG (9)	12.6 ± 2.1	9.2 ± 0.9	−3.4 (−6.4 to −0.5)	0.085	0.002	0.161	−0.40
	CG (10)	9.0 ± 0.4	7.6 ± 0.3	−1.4 (−1.8 to −0.7)				
Flexibility (cm)	IG (9)	11.3 ± 2.4	15.7 ± 2.3	4.4 (2.5–6.2)	0.060	<0.001	0.139	0.24
	CG (10)	19.6 ± 3.1	21.9 ± 3.0	2.3 (0.4–4.3)				

* Different from baseline (*p* < 0.05); m, meters; rep, repetitions; s, seconds; cm, centimeters; IG, intervention group; CG, control group; CI, confidence interval; different letters (a, b) indicate difference between groups.

The symptoms related to functionality (PCFS, MMRC, and Tilburg scale) are presented in Table 4. The results on the PCFS and MMRC scales showed improvements in both groups in the PP analysis; however, in the ITT analysis, only PCFS showed improvements in both groups. The results in the Tilburg scale, physical and general domain, showed improvement in both groups for both analyses (ITT and PP).

**Table 4:** Functionality-related symptoms before and after the intervention—scales (*n* = 40).

Outcomes	Group (*n*)	Baseline	24 weeks	Mean difference (95% CI)	*p* group	*p* time	*p* interaction	ES
Intention to treat (ITT)								
Functional status—PCFS	IG (21)	2.4 ± 0.2	1.6 ± 0.3	−0.8 (−1.4 to 0.0)	0.882	0.019	0.330	−0.45
	CG (19)	2.1 ± 0.2	1.8 ± 0.3	−0.3 (−0.7 to 0.1)				
Dyspnea—MMRC	IG (21)	1.0 ± 0.2	0.8 ± 0.3	−0.2 (−0.7 to 0.4)	0.336	0.090	0.265	0.33
	CG (19)	1.5 ± 0.3	0.9 ± 0.1	−0.6 (−1.2 to 0.0)				
Frailty—Tilburg (physical domain)	IG (21)	3.3 ± 0.4	1.9 ± 0.4	−1.4 (−2.3 to −0.4)	0.272	<0.001	0.935	−0.04
	CG (19)	3.9 ± 0.5	2.6 ± 0.4	−1.3 (−2.5 to −0.1)				
Frailty—Tilburg (psychological domain)	IG (21)	2.0 ± 0.3	1.4 ± 0.3	−0.6 (−1.4 to 0.2)	0.122	0.214	0.400	−0.29
	CG (19)	2.4 ± 0.3	2.3 ± 0.3	−0.1 (−0.9 to 0.6)				
Frailty—Tilburg (social domain)	IG (21)	0.8 ± 0.1	0.8 ± 0.1	0.0 (−0.3 to 0.4)	0.822	0.747	0.451	0.30
	CG (19)	0.9 ± 0.1	0.7 ± 0.1	−0.2 (−0.4 to 1.0)				
Frailty—Tilburg (general)	IG (21)	6.1 ± 0.7	4.1 ± 0.7	−2.0 (−3.2 to −0.6)	0.056	0.001	0.715	−0.15
	CG (19)	7.2 ± 0.7	5.7 ± 0.9	−1.5 (−3.1 to 0.0)				
Per protocol (PP)								
Functional status—PCFS	IG (9)	3.2 ± 0.2	1.8 ± 0.4	−1.4 (−2.4 to −0.2)	0.120	0.004	0.086	−1.25
	CG (12)	2.0 ± 0.3	1.8 ± 0.3	−0.2 (−0.6 to −0.0)				
Dyspnea—MMRC	IG (9)	1.2 ± 0.2	0.7 ± 0.4	−0.5 (−1.2 to 0.3)	0.345	0.041	0.601	0.16
	CG (12)	1.7 ± 0.4	1.0 ± 0.2	−0.7 (−1.5 to 0.0)				
Frailty—Tilburg (physical domain)	IG (9)	3.2 ± 0.3	2.0 ± 0.5	−1.2 (−2.2 to −0.2)	0.105	<0.001	0.536	0.39
	CG (12)	4.5 ± 0.6	2.6 ± 0.5	−1.7 (−3.0 to −0.4)				
Frailty—Tilburg (psychological domain)	IG (9)	1.4 ± 0.3	1.3 ± 0.3	−0.1 (−0.5 to 0.3)	0.055	0.536	0.812	0.06
	CG (12)	2.5 ± 0.5	2.3 ± 0.3	−0.2 (−1.3 to 0.7)				
Frailty—Tilburg (social domain)	IG (9)	1.1 ± 0.1	1.0 ± 0.2	−0.1 (−0.3 to 0.0)	0.406	0.402	0.652	0.16
	CG (12)	0.8 ± 0.1	0.7 ± 0.1	0.1 (−0.3 to 0.2)				
Frailty—Tilburg (general)	IG (9)	5.7 ± 0.6	4.3 ± 0.9	−1.4 (−2.7 to −0.1)	0.110	0.002	0.350	0.25
	CG (12)	7.8 ± 1.0	5.7 ± 0.9	−2.1 (−4.4 to −0.6)				

* Different from baseline (*p* < 0.05); m, meters; rep, repetitions; s, seconds; cm, centimeters; IG, intervention group; CG, control group; CI, confidence interval; PCFS, post-COVID-19 functional status; MMRC, medical research council; different letters indicate difference between groups.

**Figure 4:**
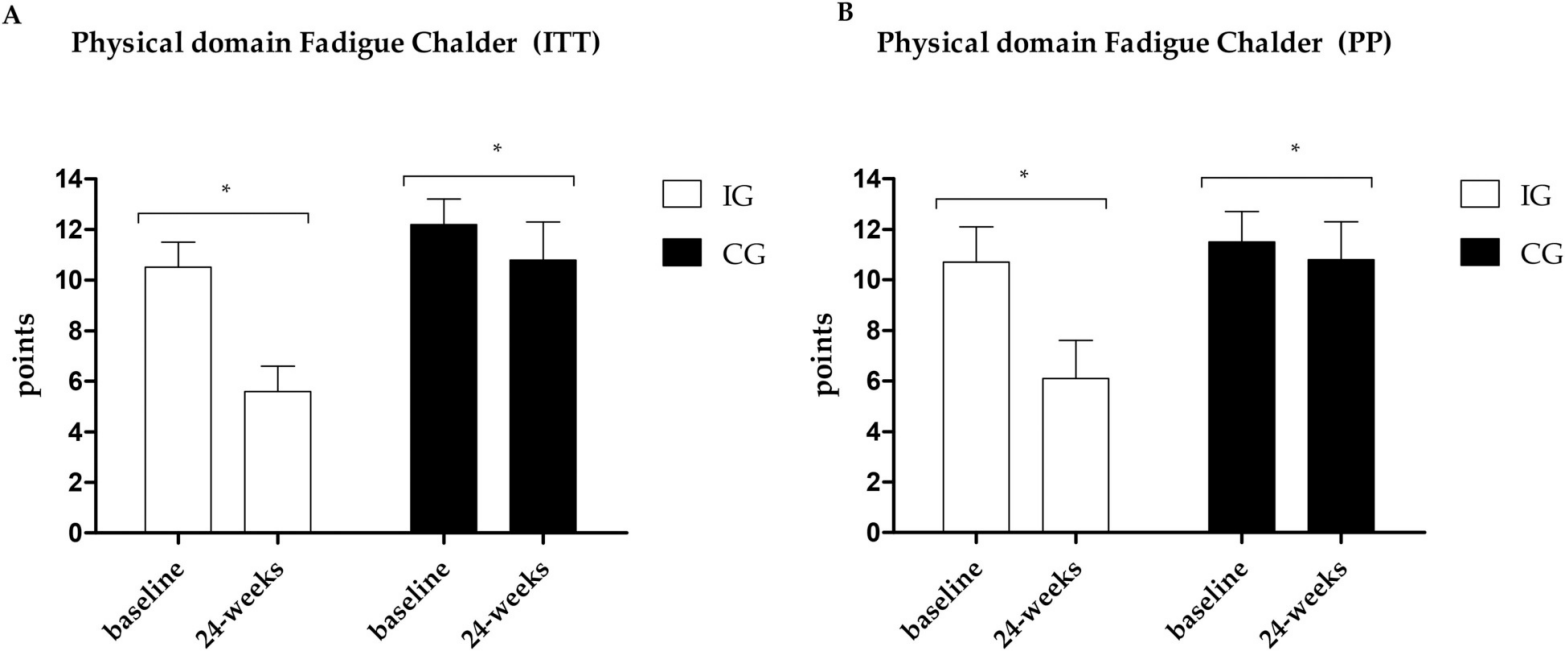
**(A)** Results of the Chalder scale—physical domain in the PP analysis. **(B)** Results of the Chalder scale—physical domain in the ITT analysis. *Different values between baseline and 24 weeks.

**Figure 5:**
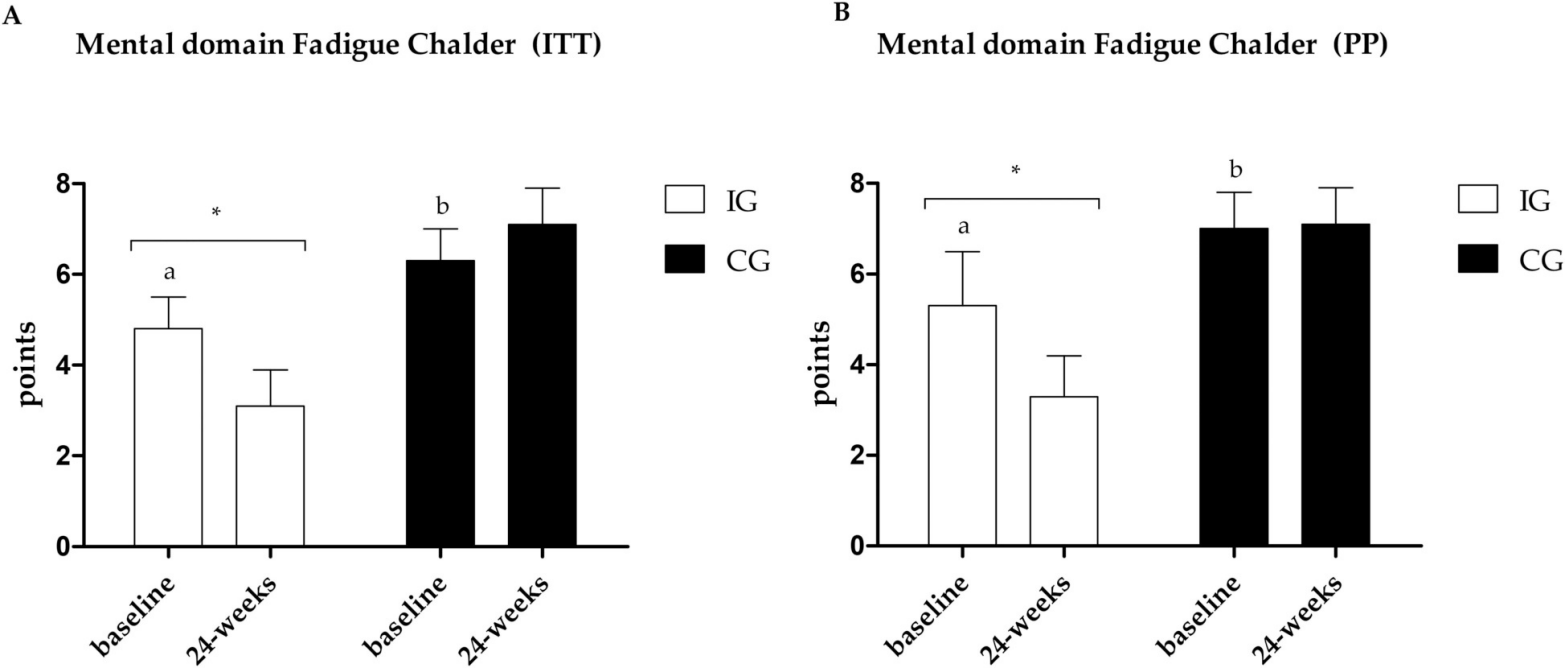
**(A)** Results of the Chalder scale—mental domain in the PP analysis. **(B)** Results of the Chalder scale—mental domain in the ITT analysis. *Different values between baseline and 24 weeks. Different letters indicate differences between groups at baseline.

**Figure 6:**
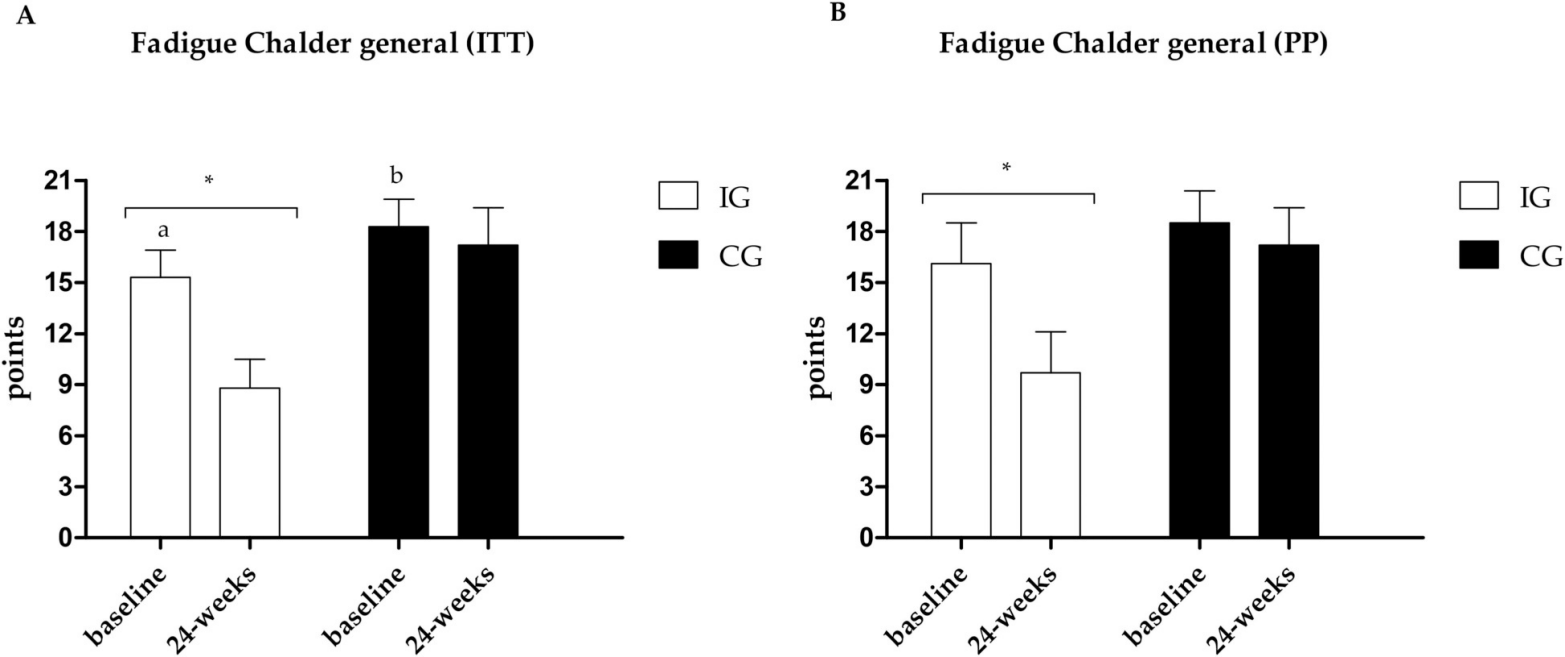
**(A)** Results of the Chalder scale—general fatigue in the PP analysis. **(B)** Results of the Chalder scale—general fatigue in the ITT analysis. *Different values between baseline and 24 weeks. Different letters indicate differences between groups at baseline.

Fatigue data are presented in Figures 4–6. Regarding the physical domain of the Chalder Fatigue Scale, both groups showed improvement in both the ITT and PP analyses. For the Chalder scale in the mental domain, both analyses showed improvement only in the IG (ITT: ES: −0.74; 95% CI: −0.5 to 3.5; *p* = 0.005/PP: ES: −0.63; 95% CI: −3.6 to 0.7; *p* = 0.017) with differences between groups at post-intervention showing better values in the IG. In the Chalder scale in the general domain, both analyses showed improvement only in the IG (ITT: ES: −0.70; 95% CI: −9.4 to −3.5; *p* = 0.026/PP: ES: −0.72; 95% CI: −11.0 to −1.6; *p* = 0.047) with differences between groups at post-intervention showing better values in the IG in the ITT analysis.

### 3.3 Body composition outcomes

The results regarding anthropometric, body composition, and PhA outcomes are presented in Table 5. In the ITT analysis, no significant changes were identified (all *p* > 0.05).

**Table 5:** Body composition before and after the intervention (*n* = 40).

Outcomes	Group (*n*)	Baseline	24 weeks	Mean difference (95% CI)	*p* group	*p* time	*p* interaction	ES
Intention to treat (ITT)								
BMI (kg/m^2^)	IG (21)	31.3 ± 1.5	31.3 ± 1.3	0.0 (−2.2 to 2.2)	0.144	0.210	0.203	0.33
	CG (19)	29.9 ± 1.3	27.7 ± 1.2	−2.1 (−4.7 to 0.3)				
Body fat (%)	IG (21)	36.0 ± 1.8	37.2 ± 2.0	1.2 (−1.0 to 3.3)	0.863	0.199	0.933	−0.02
	CG (19)	35.5 ± 1.7	36.8 ± 2.3	1.3 (−1.7 to 4.3)				
Fat mass (kg)	IG (21)	32.0 ± 3.0	32.5 ± 2.7	0.5 (−4.1 to 5.0)	0.258	0.713	0.713	0.17
	CG (19)	29.4 ± 2.1	27.7 ± 2.2	−1.6 (−5.7 to 2.4)				
Fat-free mass (kg)	IG (21)	54.2 ± 2.8	53.0 ± 2.3	−1.2 (−5.6 to 3.3)	0.352	0.069	0.248	0.32
	CG (19)	52.9 ± 2.6	47.7 ± 3.2	−5.2 (−10.4 to 0.0)				
Total lean mass (kg)	IG (21)	51.6 ± 2.7	50.6 ± 2.2	−1.0 (−5.3 to 3.2)	0.300	0.071	0.234	0.33
	CG (19)	50.2 ± 2.5	45.1 ± 3.0	−5.0 (−10.0 to 0.0)				
Leg lean mass (kg)	IG (21)	16.2 ± 0.8	16.6 ± 0.8	0.4 (−0.9 to 1.7)	0.395	0.434	0.130	0.43
	CG (19)	16.1 ± 0.8	14.8 ± 1.0	−1.3 (−2.9 to 0.4)				
Arm lean mass (kg)	IG (21)	6.5 ± 1.0	5.5 ± 0.3	−1.0 (−3.1 to 1.0)	0.171	0.116	0.746	−0.09
	CG (19)	5.4 ± 0.3	4.7 ± 0.4	−0.7 (−1.3 to 0.0)				
Visceral adipose tissue (g)	IG (21)	823.0 ± 74.1	920.0 ± 68.6	96.9 (−4.3 to 198.2)	0.480	0.107	0.054	0.45
	CG (19)	831.5 ± 83.5	762.8 ± 93.0	−68.7 (−203.5 to 66.0)				
Waist (cm)	IG (21)	98.0 ± 2.9	98.5 ± 3.0	0.4 (−4.0 to 4.8)	0.224	0.320	0.221	0.33
	CG (19)	95.5 ± 3.0	91.4 ± 3.3	−4.1 (−9.9 to 1.6)				
WHR	IG (21)	0.58 ± 0.16	0.59 ± 0.18	0.01 (−0.02 to 0.02)	0.199	0.394	0.279	0.28
	CG (19)	0.57 ± 0.01	0.55 ± 0.02	−0.01 (−0.51 to 0.01)				
Phase angle (°)	IG (21)	5.40 ± 0.20	5.40 ± 0.20	0.00 (−0.32 to 0.31)	0.937	0.937	0.658	0.12
	CG (19)	5.47 ± 0.18	5.36 ± 0.22	−0.11 (−0.49 to 0.26)				
Per protocol (PP)								
BMI (kg/m^2^)	IG (9)	31.7 ± 1.9	31.5 ± 2.0	−0.2 (−0.6 to 0.2)	0.071	0.283	0.015	−0.15
	CG (11)	27.1 ± 1.1	27.7 ± 1.2*	0.6 (0.1–1.0)				
Body fat (%)	IG (9)	37.2 ± 2.9	36.7 ± 2.9 *	−0.5 (−1.0 to −0.1)	0.869	0.535	0.021	−0.16
	CG (11)	35.9 ± 2.2	36.8 ± 2.2	0.9 (−0.2 to 2.0)				
Fat mass (kg)	IG (9)	31.6 ± 3.5	31.0 ± 3.5	−0.6 (−1.3 to 0.1)	0.300	0.173	<0.001	−0.22
	CG (11)	26.3 ± 2.0	27.7 ± 2.2*	1.4 (0.5–2.5)				
Fat-free mass (kg)	IG (9)	51.2 ± 3.4	51.4 ± 3.5	0.2 (−0.5 to 0.8)	0.389	0.225	0.427	−0.06
	CG (11)	47.0 ± 2.8	47.7 ± 3.2	0.7 (−0.5 to 2.0)				
Total lean mass (kg)	IG (9)	48.9 ± 3.2	49.2 ± 3.4	0.3 (−0.4 to 1.0)	0.328	0.164	0.574	−0.04
	CG (11)	44.4 ± 2.6	45.1 ± 3.0	0.7 (−0.5 to 2.0)				
Leg lean mass (kg)	IG (9)	15.7 ± 1.0	16.0 ± 1.1	0.3 (−0.1 to 0.8)	0.350	0.034	0.503	−0.09
	CG (11)	14.2 ± 0.8	14.8 ± 1.0	0.6 (−0.1 to 1.3)				
Arm lean mass (kg)	IG (9)	5.2 ± 0.4	5.3 ± 0.4	0.1 (0.1 to 0.3)	0.360	0.013	0.731	0.02
	CG (11)	4.6 ± 0.4	4.7 ± 0.4	0.1 (−0.1 to 0.3)				
Visceral adipose tissue (g)	IG (9)	961.6 ± 127.5	902.4 ± 102.7	−59.2 (−139.4 to 21.0)	0.176	0.854	0.015	−0.32
	CG (11)	694.0 ± 105.9	762.8 ± 93.0*	68.8 (4.6–132.9)				
Waist (cm)	IG (9)	97.7 ± 4.5	97.5 ± 4.9	−0.2 (−3.3 to 2.9)	0.210	0.385	0.261	−0.15
	CG (11)	89.7 ± 3.0	91.4 ± 3.4	1.7 (0.3 to 3.1)				
WHR	IG (9)	0.60 ± 0.02	0.60 ± 0.02	0.00 (−0.02 to 0.02)	0.118	0.419	0.301	0.28
	CG (11)	0.55 ± 0.02	0.55 ± 0.02	0.00 (0.00–0.02)				
Phase angle (°)	IG (9)	5.02 ± 0.31	5.14 ± 0.26	0.11 (−0.16 to 0.39)	0.512	0.185	0.898	−0.06
	CG (10)	5.26 ± 0.20	5.36 ± 0.22	0.09 (−0.05 to 0.25)				

* Different from baseline (*p* < 0.05); BMI, body mass index; CG, control group; CI, confidence interval; ES, effect size; IG, intervention group; WHR, waist–height ratio.

The PP analysis identified that the CG increased the BMI (mean difference = 0.6 kg/m^2^; *p* = 0.018), fat mass (mean difference = 1.4 kg; *p* = 0.004), and the amount of visceral adipose tissue (mean difference = 68.8 g; *p* = 0.036). For the fat percentage, the IG showed a reduction (mean difference = −0.5%; *p* = 0.030). Both groups showed increased leg and arm lean mass in the PP analysis.

### 3.4 Physical activity and sedentary behavior

There were no significant differences between baseline and post-intervention in the minutes spent in light physical activity per week (IG: pre 2,056.2 ± 128.1; post 1,991.6 ± 164.0; CG: pre 2,303.9 ± 182.2; post 2,299.6 ± 174.9; *p* = 0.811) and moderate/vigorous physical activity (IG: pre 171.4 ± 26.5; post 152.3 ± 36.1; CG: pre 133.5 ± 28.1; post 112.5 ± 36.5; *p* = 0.970), nor in the minutes spent in sedentary time (IG: pre 4,646.2 ± 340.7; post 4,744.5 ± 286.6; CG: pre 4,089.8 ± 156.9; post 4,307.7 ± 204.4; *p* = 0.671).

### 3.6 Pulmonary function

The pulmonary function results are summarized in Table 6. The ITT analysis revealed no significant differences between baseline and post-intervention values (all *p* > 0.05). The PP analysis could not be performed due to a substantial loss of participants in the post-intervention pulmonary function assessments.

**Table 6:** Pulmonary function in the pre- and post-intervention moments (*n* = 34).

Outcomes	Group (*n*)	Baseline	24 weeks	Mean difference (95% CI)	*p* group	*p* time	*p* interaction	ES
Intention to treat (ITT)								
VC (L)	IG (18)	3.68 ± 0.20	3.82 ± 0.24	0.14 (−0.26 to 0.53)	0.910	0.683	0.530	0.22
	CG (16)	3.80 ± 0.14	3.77 ± 0.24	−0.03 (−0.35 to 0.30)				
DLCO (mL/min/mmHg)	IG (18)	18.45 ± 1.21	18.48 ± 1.12	0.03 (−2.67 to 2.72)	0.653	0.698	0.674	0.15
	CG (16)	18.21 ± 0.87	17.53 ± 1.16	−0.68 (−2.56 to 1.22)				
VA (L)	IG (18)	4.57 ± 0.20	4.61 ± 0.20	0.04 (−0.35 to 0.42)	0.650	0.605	0.436	0.25
	CG (16)	4.80 ± 0.19	4.62 ± 0.24	−0.18 (−0.56 to 0.20)				
KCO (mL/mmHg/min/L)	IG (18)	3.99 ± 0.13	3.99 ± 0.15	0.00 (−0.33 to 0.32)	0.351	0.941	0.952	0.02
	CG (16)	3.81 ± 0.15	3.80 ± 0.21	0.01 (−0.35 to 0.32)				
DLCO (%)	IG (18)	76.68 ± 3.08	78.57 ± 3.00	1.88 (−5.35 to 9.12)	0.962	0.405	0.928	−0.03
	CG (16)	76.70 ± 3.36	79.05 ± 5.99	2.34 (−4.50 to 9.19)				
VA (%)	IG (18)	89.84 ± 2.30	88.49 ± 5.03	−1.35 (−10.48 to 7.77)	0.606	0.909	0.735	−0.14
	CG (16)	91.49 ± 3.76	92.16 ± 4.74	0.66 (−6.68 to 8.02)				
KCO (%)	IG (18)	98.14 ± 2.83	98.10 ± 3.28	−0.03 (−7.34 to 7.27)	0.453	0.874	0.884	0.06
	CG (16)	95.25 ± 3.50	94.40 ± 4.81	−0.85 (−8.98 to 7.28)				

IG, intervention group; CG, control group; CI, confidence interval; VC, vital capacity; DLCO, diffusing capacity of the lungs for carbon monoxide; VA, alveolar volume; KCO, lung transfer coefficient for carbon monoxide.

## 4 Discussion

To the best of our knowledge, this is the first randomized controlled clinical trial to examine the effects of a supervised multicomponent training program, encompassing balance, aerobic, and strength exercises, lasting 24 weeks in critically debilitated patients with COVID-19. Our results indicate the non-superiority of the IG in the intention-to-treat analysis concerning the primary outcome; however, the IG demonstrated superiority in mental and general fatigue. In the per-protocol analysis, the IG showed superiority in several outcomes, including the primary outcome and body composition outcomes, demonstrating that high adherence to the program brought greater benefits to the participants. No significant differences were observed in physical activity levels or sedentary behavior and pulmonary function.

The primary outcome, 6MWT, showed an improvement in both groups in the ITT analysis (IG: 35 m; CG: 32 m), but in the PP analysis, only the IG had a significant change (IG: 88 m; CG: 16 m). Despite the difference between the groups at the baseline of the PP analysis, the moderate effect size (ES: 0.61) indicates superiority of the IG and significant clinical relevance of using multicomponent training in post-COVID rehabilitation. Our findings reinforce the extant literature of evidence indicating functional improvements, especially using the 6MWT, after different physical training models in the post-COVID-19 population (57–59).

In the context of the 6MWT, it's worth mentioning that the difference between pre- and post-intervention aligns with a study by McDonald et al. (59), which indicates an approximate 30 m difference as protective for various comorbidities. Particularly in the results of the PP analysis of the present study, an improvement of approximately 50 m is observed at the lower limit of the CI (95%).

The improvement found in the CG in functional capacity outcomes may possibly be explained by the cumulative effect of the natural course of post-illness recovery, even up to a year after infection. However, when we examined only the patients who adhered to the training in the IG, we found significant results in the 6MWT and TUG-m speed in the IG, with moderate effect sizes (0.61 and −0.51, respectively), demonstrating enhanced functional rehabilitation through the practice of supervised physical exercises. However, adherence to the intervention is required.

The improvement in the IG in TUG-m speed in PP analysis can be explained by some factors, such as the specific balance training carried out in Phase 1 of the program. The best values for this outcome may imply a better quality of life, as worse TUG scores are associated with a higher BMI, more comorbidities, and worse perception of physical health (60).

The positive results in post-COVID functional status (PCFS), mainly in PP analyses, with a large ES (1.25), reinforce the positive effects of multicomponent training in recovering the functional capacity of individuals after COVID-19 infection. In the MMRC, both groups showed improvements in the PP analysis. Dyspnea is one of the most reported persistent symptoms during and after COVID-19 infection (61).

In the Tilburg scale, improvements were found in the physical and general domain in both groups in the ITT and PP analysis, and even in the case of adults and elderly people (average age: 52.25 ± 13.00 years), both groups started participation in the study with values >5 in the general domain, indicating a state of fragility (62). Positively, after 24 weeks, in both analyses, the IG left the frailty zone, indicating scores <5, which did not occur in the CG, which continued in the frailty zone with scores >5 despite the improvement.

Fatigue is one of the most prevalent symptoms in acute post-COVID-19 or long-term COVID patients (8–10), and it's the most related symptom in the sample during the post-acute phase. An improvement was observed only in the IG in both analyses (ITT and PP) in the mental and general domains. In the physical domain, the ITT analysis shows improvement in both groups with a moderate ES between groups (ES: −0.74), and the PP analysis brings a large ES (ES: −0.89). Fatigue is associated with a decline in quality of life, a reduction in the ability to perform activities of daily living, and a reduction in the ability to produce maximum strength or power (63); therefore, reducing the levels of fatigue found can directly help in improving fatigue, quality of life, and functional capacity of individuals.

The evidence indicates that muscle damage resulting from mitochondrial changes, inflammation, and capillary injury in muscle biopsies could be one of the possible causes of post-COVID-19 (16–18), so we could expect the recovery of both to occur in parallel. However, we did not present results for body composition, possibly due to the multifactorial nature that fatigue presents in COVID-19 survivors, and especially due to the possible contribution of psychological factors that seem to have a great influence in longer periods after the acute phase of COVID-19 (64). Additionally, 1 year after hospital discharge, it was found that overweight and obese individuals admitted to the ICU had increased lean body mass, but 62% of these patients had fatigue (65).

In addition to the prior recovery of lean mass that could possibly have already occurred with the participants, according to the previously mentioned study, the low weekly training volume, an important factor for the hypertrophy process (66, 67), could possibly explain the lack of results, in general, in the ITT analysis for body composition parameters. When we adjusted the analysis to only individuals who participated in >70% of the training sessions, we could see some small changes, mainly regarding protection against increased body fat (ES: −0.22) and visceral adipose tissue (ES: −0.32), which becomes important as evidence shows that there is a trend toward an increase in body fat post-COVID-19 (68–70). It is also important to highlight that the CG did not follow a traditional control, as there was a meeting with exercise professionals in which the benefits of physical activity, the domains of physical activity, and recommendations for weekly practice were discussed, in addition to the delivery of two chapters of the Brazilian Guide of Physical Activity (39).

This study found no significant differences in lung function parameters between baseline and post-intervention in the ITT analysis. Notably, both groups presented DLCO mean values <80% of the predicted values at both assessments, reflecting persistent impairment in pulmonary diffusion capacity. These findings are consistent with prior systematic reviews and meta-analyses indicating that impaired DLCO is the most frequently observed abnormality in pulmonary function tests between 6 and 12 months after recovery from COVID-19 (71), with a reported prevalence of 43% (95% CI: 22%–65%) beyond 6 months post-hospital discharge (72). Unfortunately, the considerable loss of participants in post-intervention lung function assessments precluded the performance of the PP analyses, preventing a conclusive evaluation of the effects of supervised exercise on pulmonary outcomes.

As strengths of this study, it's worth noting that the program demonstrated safety, a currently debated aspect in randomized controlled trials of post-COVID-19 rehabilitation programs (33). The program proved to be well tolerated, with few sample losses (*n* = 8), and a reasonable average participation (60.9%). Despite the low number of individuals from the IG included in the per-protocol analysis (*n* = 9), the high adherence among these patients stands out, with a frequency of 81.61%. Additionally, a decrease in internal load and an increase in external load over time demonstrate a possible adaptation to training and improvement in exercise tolerance.

Another point is that there is no study to date that has reported outcomes resulting from a 24-week intervention. Furthermore, the program was administered approximately a year after participants contracted COVID-19, and most intervention studies took place over a period of no more than 6 months. Therefore, the positive changes observed, particularly in the per-protocol analysis, underscore the significance of continued efforts to improve functional outcomes and body composition even a year after infection.

The study presents some limitations, such as the limited sample size in the per-protocol analysis, where a significant number of participants did not reach the 70% session attendance frequency. To mitigate these limitations, in addition to monitoring attendance and frequently contacting absent participants, instructors offered the opportunity for makeup training sessions in Phase 1 of the study, as well as alternative scheduling options for sessions, in cases requested by participants.

As clinical applications, this study presents a low-cost and easy-to-apply training protocol that can be easily replicated in different scenarios. In addition, it differs from most investigations with the post-COVID-19 infection population, mainly in methodological rigor, both in clinical methodological aspects such as randomization, blinding, and control, and in the control of the training prescription that combined individualized prescriptions in three different physical qualities and requires a minimum use of equipment, in a frequency of just two weekly sessions.

## 5 Conclusion

We concluded that the 24-week multicomponent physical training is effective in reducing one of the most prevalent persistent symptoms in the post-COVID condition, fatigue and mental fatigue, in individuals infected with SARS-CoV-2. However, the results are not superior to the recommendations for structured physical activity in outcomes related to functional capacity, body composition, and physical activity levels among individuals who did not reach the 70% session attendance frequency.

## Data Availability

The raw data supporting the conclusions of this article will be made available by the authors, without undue reservation.
